# A Very Rare Variant of Cerebral Autosomal Dominant Arteriopathy With Subcortical Infarcts and Leucoencephalopathy (CADASIL) on the gnomAD Database With Variable Phenotypic Expression

**DOI:** 10.7759/cureus.83725

**Published:** 2025-05-08

**Authors:** Ikechukwu Chukwuocha, Solomon Eigbe, Di Liang, Baig Al-Moyeed

**Affiliations:** 1 Neurology, The Royal Wolverhampton NHS Trust, Wolverhampton, GBR

**Keywords:** cerebral autosomal dominant arteriopathy with subcortical infarcts and leukoencephalopathy (cadasil), neuroimaging findings, notch3 gene, small vessel ischaemic disease, stroke

## Abstract

Cerebral autosomal dominant arteriopathy with subcortical infarcts and leucoencephalopathy (CADASIL) is an important genetic cause of stroke and vascular dementia, which may also demonstrate variable phenotypic expression. The causative mutation is in the NOTCH3 gene, which maps to chromosome 19 and is largely expressed in the vascular smooth muscle cells of small cerebral blood vessels. The predominant clinical manifestations of this disease include migraines, subcortical ischaemic events, mood disturbances, apathy, and cognitive decline. We herein describe a 46-year-old man who presented to the emergency department with slurred speech, right-sided facial drop, and right lower limb incoordination. Three years before the presentation, he had episodes of right-sided leg stiffness and visual disturbances, which were suspected to be a demyelinating event. Physical examination confirmed increased tone in his lower limbs, worse on the right. His brain MRI showed severe, widespread white matter T2 signal abnormality. The possibility of an adult-onset leucodystrophy was entertained, and the diagnosis of CADASIL was confirmed through genetic testing, which identified a mutation in the NOTCH3 gene. This paper outlines the aetiopathogenesis, clinical presentation, investigations, and management of CADASIL.

## Introduction

Cerebral autosomal dominant arteriopathy with subcortical infarcts and leucoencephalopathy (CADASIL) is the most important heritable cause of adult vascular dementia and stroke and results from mutation of the NOTCH3 gene located on chromosome 19 [1]. It is an uncommon vascular disorder with an autosomal dominant pattern of inheritance [2].

The underlying pathology consists of abnormal transmembrane deposits building up on blood vessel smooth muscle cells in the brain and other organs [3], damaging these vessels, and making the patient more vulnerable to ischaemic events. This manifests as migraine with aura in certain persons, while other individuals experience transient ischaemic attacks (TIAs) or strokes that occur before the age of 60 years [1].

The clinical trajectory is quite unpredictable. While some patients experience severe symptoms as early as their 20s, others do not experience any symptoms until later in life [4]. The accumulation of these ischaemic events predisposes the patients to vascular cognitive impairment, which may eventually lead to death [5].

## Case presentation

This is a 46-year-old gentleman who presented with the complaint of a one-week right-sided facial weakness and slurred speech, which started acutely with a gradual build-up over 24 hours before plateauing with associated minor dysphagia. Before the onset of the present symptoms, he experienced what he described as an episode of imbalance, poor coordination, and visual disturbance four years earlier, which was, at the time, suspected to be an episode of demyelinating event based on multiple cerebral white matter lesions on brain MRI. He reported that he did not have any specific treatment and that he recovered fairly well.

He remained symptom-free until five months before the index presentation, when he had another episode of right leg stiffening and spasms, which remained persistent, and he felt that his right ankle and leg were not quite right as he was more off balance. He denied any new significant motor or sensory changes, cognitive deficit, headaches, sphincter abnormalities, or any other neurological symptoms; however, he was reported by his partner to have significant mood changes. He was otherwise systemically well; he takes alcohol occasionally and denied the use of illicit medications/cigarette smoking. His mother had a brain tumour in her 40s, and his father had a stroke. He has a son who is currently healthy and well.

Neurological examination was relevant for a mild right facial weakness. Reflexes were brisk and symmetrical, with a notable increase in ankle clonus. Plantar response was equivocal. Other aspects of the examination did not reveal any focal signs.

**Figure 1:**
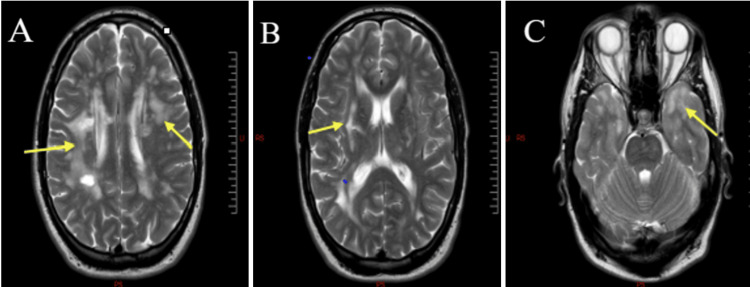
Axial T2-weighted MRI image of the index patient Presence of focal and confluent hyperintense lesions located bilaterally in the periventricular region (yellow arrows) (A), characteristic high-signal intensity of the external capsule (yellow arrow) (B), and anterior temporal pole (yellow arrow) (C)

**Figure 2:**
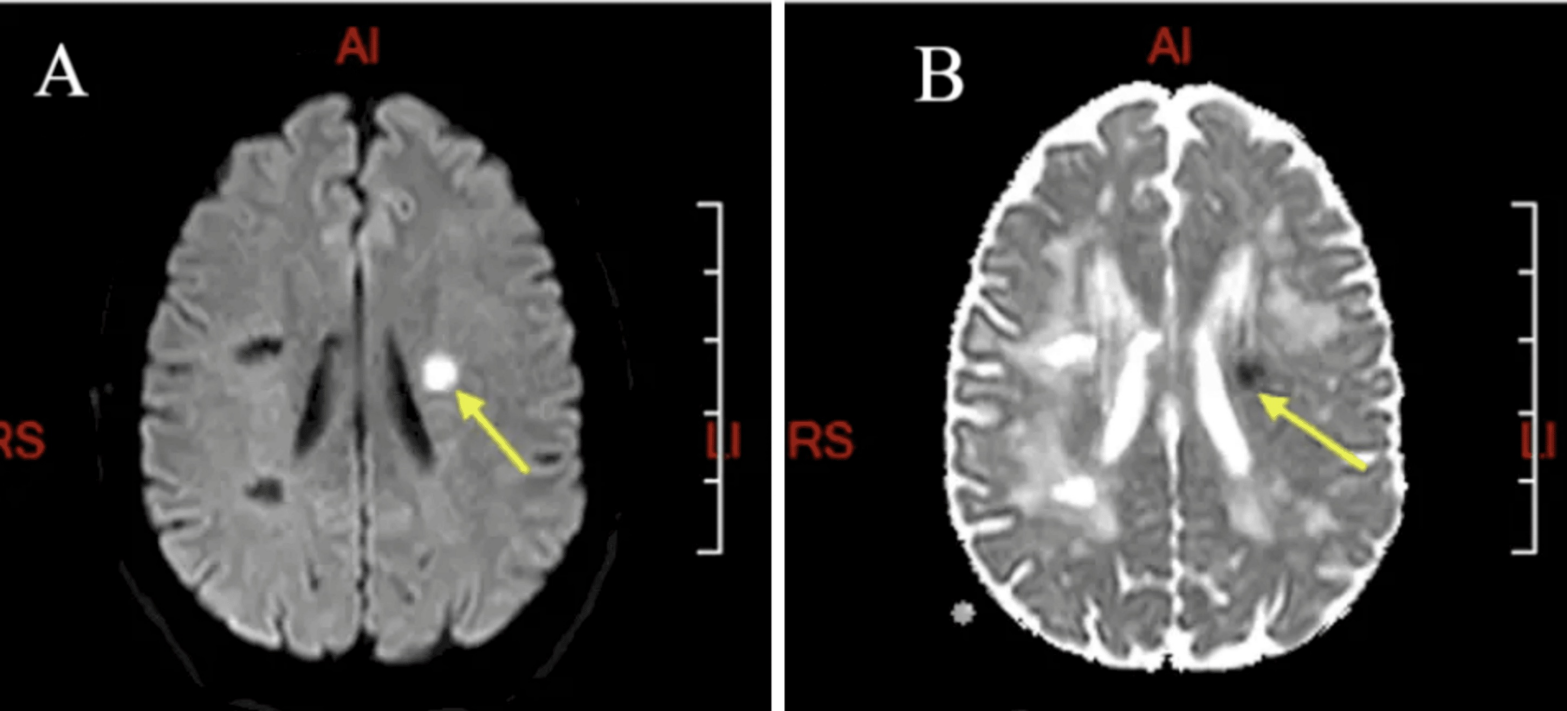
Brain MRI of the index patient: DWI and ADC sequences DWI hyperintense lesion (yellow arrow) (A), with corresponding ADC lesion (yellow arrow) (B) suggestive of acute ischaemic stroke DWI: diffusion-weighted imaging; ADC: apparent diffusion coefficient

The head CT did not show any abnormality, and head and neck vessel imaging was normal. A brain MRI revealed a marked leucoencephalopathy in the periventricular region (A), external capsule (B), and anterior temporal pole (C) (Figure 1) and radiological evidence of an acute ischaemic event (Figure 2).

A lumbar puncture was done, which revealed slightly elevated protein in CSF, and an identical oligoclonal band was seen in serum and CSF, suggesting a possible inflammatory process; these findings would increase suspicion of a demyelinating process. Screening for paraneoplastic antibodies and antibodies for demyelinating conditions such as myelin oligodendrocyte glycoprotein (MOG) antibody disease (MOGAD) and neuromyelitis optica (NMO) was negative. HIV serology and infectious and immune screen, as well as the rest of his blood work, were unremarkable. A genetic study for adult-onset leucoencephalopathy panel was positive for a mutation in the NOTCH3 gene (Table 1).

**Table 1:** Laboratory results HDL: high-density lipoprotein; LDL: low-density lipoprotein

Tests	Results	References
CSF WBC count (×10^6^/L)	0 cells	0-5
CSF RBC count (×10^6^/L)	0 cells	0
CSF total protein (g/L)	0.5	0.15-0.45
CSF glucose (mmol/L)	3.5	2.2-3.9
Complement C3 (g/L)	1.38	0.75-1.65
Complement C4 (g/L)	0.24	0.14-0.54
Cardiolipin immunoglobulin M Abs (U/mL)	6.2	0-40
Cardiolipin immunoglobulin G Abs (U/mL)	3.7	0-10
Beta-2 glycoprotein 1 IgM Abs (kU/L)	9.1	0-10
Beta-2 glycoprotein 1 IgG Abs (kU/L)	2.2	0-10
ESR (mm/hr)	2	0-20
HbA1c (mmol/mol)	31	20-41
Total cholesterol (mmol/L)	6.1	3.5-6.5
HDL cholesterol (mmol/L)	1.3	≥1
LDL cholesterol (mmol/L)	3.9	≤3
Triglycerides (mmol/L)	2.0	<1.7

He was subsequently reviewed in the outpatient neurology clinic and had complete resolution of his facial asymmetry and dysphagia, but some residual mild right leg incoordination. His Addenbrooke’s cognitive examination (ACE-III) was 94/100: attention (15/18), memory (26/26), fluency (14/14), language (23/26), and visuospatial (16/16).

He was placed on anti-platelet agents and a lipid-lowering agent as secondary prevention of his ischaemic event and referred to formal genetic counselling and review in the young-onset dementia clinic for follow-up.

## Discussion

CADASIL is a monogenic small vessel vasculopathy pathologically marked by diffuse angiopathy that is non-atherosclerotic nor amyloid, mainly affecting small- to medium-sized penetrating arteries and leptomeningeal arteries, causing recurrent neurological events [6,7]. The mutations causing this disease map to the long arm of chromosome 19 and encode the NOTCH3 receptor protein, a large extracellular domain with 34 epidermal growth factor-like repeats encoded by exons 2-24, predominantly expressed in adults by vascular smooth muscle cells (VSMCs) and pericytes [8]. It is also important to note that the short arm of the same chromosome 19 encompasses the locus for familial hemiplegic migraine.

This hereditary illness damages the brain's tiny blood vessels, reducing blood flow to specific brain regions and causing multiple ischaemic lesions with differing symptoms depending on where the lesions are located [9]. Because the disorder is inherited in an autosomal dominant fashion, disease manifestation is also anticipated in heterozygotes.

Several patients with CADASIL have been identified since the NOTCH3 mutation was discovered in 1993 with marked phenotypic variability. Common CADASIL symptoms include aura-associated migraine, TIAs, and recurrent ischaemic strokes. These can progress to cause cognitive decline exhibiting as apathy/mood swings and severe dementia, which is life-threatening [10,11]. Our patient manifested some of these characteristic symptoms, especially transient neurological episodes with accompanying negative symptoms initially mistaken for a demyelinating event, but turned out to be episodes of ischaemic events.

Ischaemic events in these patients manifest as lacunar syndromes, such as pure motor strokes, pure sensory strokes, or dysarthria-clumsy hand syndrome, or uncommonly as expressive dysphasia or visual field defects, and they almost always happen in subcortical areas. A CADASIL patient suffers, on average, two to five ischaemic events in a lifetime, often resulting in gait difficulty and ambulatory loss, urinary urgency, and pseudobulbar palsy; these were also present in this patient [12,13]. Stepwise, progressive cognitive impairment and dementia resulting from recurrent ischaemic events mostly causing deterioration in executive function and working memory/attention are the second most common clinical manifestations in CADASIL and frequently lead to disability [14]. Some patients may also present with uncommon symptoms, such as intracerebral haemorrhage, epilepsy, acute encephalopathy, territorial infarcts, parkinsonism, and deafness, which are atypical for this condition [15].

Radiologically, CADASIL is characterised by high-signal-intensity lesions in the periventricular, deep white matter, external capsule, and anterior temporal pole with multiple lacunar infarctions in the thalamus and basal ganglia, many of which were found in our patient. In multiple sclerosis (MS), active lesions are contrast-enhanced with optic neuritis, whereas in CADASIL, there is sparing of the infratentorial brain and spinal cord [16]. However, the gold standard for the diagnosis of CADASIL is currently genetic testing that identifies the distinctive cysteine-altering mutations in the NOTCH3 gene; heterozygous mutations in the NOTCH3 (NM_000435.2)c.457C>T p(Arg153Cys) were detected in our patient following his genetic study. This mutation was earlier reported to have affected only 42 patients and 32 families [17].

CADASIL exhibits pathological distinctive characteristics of an arteriopathy that appears as granular osmiophilic material (GOM) deposits in the plasma membranes of pericytes and VSMCs, as well as VSMCs that degenerate [18]. Where the results of the genetic studies do not correlate with suggestive clinical and radiological findings for this condition, it is advised to have a skin biopsy done, which may reveal these distinctive findings in VSMCs of small blood vessels [19].

Currently, CADASIL has no known cure or effective treatment. Affected people and their families can benefit from supportive care, which includes genetic counselling, emotional support, and practical assistance. Depending on how often the symptoms occur, migraines should be treated both symptomatically and preventively. Treatment should be given for any coexisting conditions such as obstructive sleep apnoea, diabetes, high cholesterol, or hypertension. People with CADASIL should abstain from smoking since it raises their risk of stroke. Antiplatelets such as low-dose aspirin or clopidogrel can be used as a secondary preventive measure if an ischaemic event occurs [20,21]. Anticoagulants are not advised due to the possibility of brain haemorrhage, and the use of vasoconstrictor medications to treat migraine attack symptoms in this disease state is discouraged due to the possibility of ischaemia caused by these molecules [22].

## Conclusions

This case demonstrates how easily rare genetic diseases such as CADASIL are missed, especially in the absence of a strong family history and confounded with other causes of focal neurologic deficit like MS. A second ischaemic event in an otherwise young person with weak cardiovascular risk factors should increase suspicion for CADASIL. Although there are limited treatment options, patients can benefit from secondary prevention treatment and genetic counselling. Early referral to a young-onset dementia clinic will provide access to specialised support and help them to plan for the future.
